# Evaluation of children's pain expression and behavior using audio visual distraction

**DOI:** 10.1002/cre2.407

**Published:** 2021-02-23

**Authors:** Alicia Delgado, Soo‐Min Ok, Donald Ho, Tyler Lynd, Kyounga Cheon

**Affiliations:** ^1^ Department of Pediatric Dentistry University of Alabama at Birmingham Birmingham Alabama USA; ^2^ Department of Oral Medicine, Dental and Life Science Institute, Dental Research Institute Pusan National University Dental Hospital Yangsan South Korea

**Keywords:** audio‐visual distraction, children's behavior guidance, pain rating scales, pediatric dental clinic

## Abstract

**Objectives:**

Dental anxiety distresses children and their families with consequent poor oral health and costly pediatric dental services. Children's behaviors could be modified using a distraction technique for improved dental treatment. The study evaluates the effects of an audio‐visual distraction on children's behaviors and pain expressions during dental treatment.

**Material and Methods:**

One hundred healthy children, between 4 and 6 years of age, were randomly assigned to one of two groups: audio visual distraction (AVD, *N* = 61) group and control (CTR, *N* = 39) group. The pre and post pain expression was collected using a faces pain rating scale from the participated children. Children's behavior was evaluated using the Frankl behavior rating scale by the assigned dentist. Data was analyzed using chi‐squared tests and analysis of variance.

**Results:**

The AVD group demonstrated more “definitely positive” behavior (91.8%) compared to the CTR group (35.9%) based on the Frankl scale evaluation from pre‐ and post‐treatment (*p* < 0.0001). The pain rating scale did not demonstrate a significant difference in post‐treatment pain scales (*p* = 0.2073) or changes in pain (*p* = 0.1532) between the AVD group and CTR group.

**Conclusions:**

The AVD is an effective distraction tool for young children during dental treatment regardless of child's subjective pain expression.

## 1 INTRODUCTION

The prevalence of dental anxiety and fear in pediatric dental patients varies from 5% to 20% in different populations (Klingberg & Broberg, 2007). It is reported that one‐fifth of the adult population suffers from dental anxiety, and half of them have described that they developed a fear for dental treatment during their childhood dental treatments (Locker et al., 1999). Studies have shown that the development of dental anxiety and fear in children is strongly associated with negative dental experiences resulting from painful and uncomfortable dental procedures (Locker et al., 1999; Townend et al., 2000). In addition, parental attitude or perception has a significant effect onto developing child's dental anxiety (Kyritsi et al., 2009). Numerous studies reported that anxious children have higher rates of dental caries and associate with deferred or canceled appointments (Klingberg & Broberg, 2007; Sohn & Ismail, 2005).

In order to encourage children to comply with dental clinical visits, a series of behavior guidance techniques to improve children's behavior has been suggested (AAPD, 2020). Basic behavior management techniques can be provided as a fundamental method using tell‐show‐do (TSD), voice control, distraction, and positive reinforcement in most children effectively. Further, advanced behavioral management techniques, including protective stabilization, sedation, and general anesthesia are considered for implementation during the dental procedure to patients who are in a mentally, physically, or medically challenged situation (Foreman, 1988). Studies have demonstrated that appropriate behavior guidance techniques could lead to decreased medication intake, increased patient safety, and reduced side effects (Foreman, 1988; Greenbaum & Melamed, 1988). Notably, non‐restrained behavior guidance techniques may be preferred by parents and children (Jindal & Kaur, 2011). Therefore, the current trend of parenting demands development of distractive behavior guidance using audiovisual equipment, hypnosis, and music (El‐Sharkawi et al., 2012; Hoge et al., 2012). Distractive behavior guidance is the technique of diverting the patient's attention from what could be perceived as an unpleasant procedure (AAPD, 2020). Filcheck et al. reported that the display of attention‐grabbing videotaped material had an effect in distracting the children from the feared stimuli, and it was considered as one of the most attractive methods for modifying children's behavior during dental treatment (Filcheck et al., 2005). Among the non–invasive distractive behavior guidance, audiovisual distraction (AVD) is being utilized for children who watch and listen to movies during a stressful procedure. Numerous studies demonstrated the efficacy of AVD using video eyeglasses in managing distress and reducing fear and anxiety in children during dental treatments (Al‐Khotani et al., 2016; Nuvvula et al., 2015). Studies found that audiovisual eyeglasses effectively reduced reported pain during local anesthetic injections (El‐Sharkawi et al., 2012) and that audiovisual eyeglasses were a successful distraction technique during dental treatment in children (Ram et al., 2010). The null hypothesis for the study is that there is no difference in pain expression and behavior change of children between the AVD group and the CTR group. Therefore, the purpose of this study was to evaluate the effectiveness of overhead movie devices on pain expression and behavior in children, 4–6 years old during dental treatment.

## 2 METHODS

### 2.1 Participants

**FIGURE 1:**
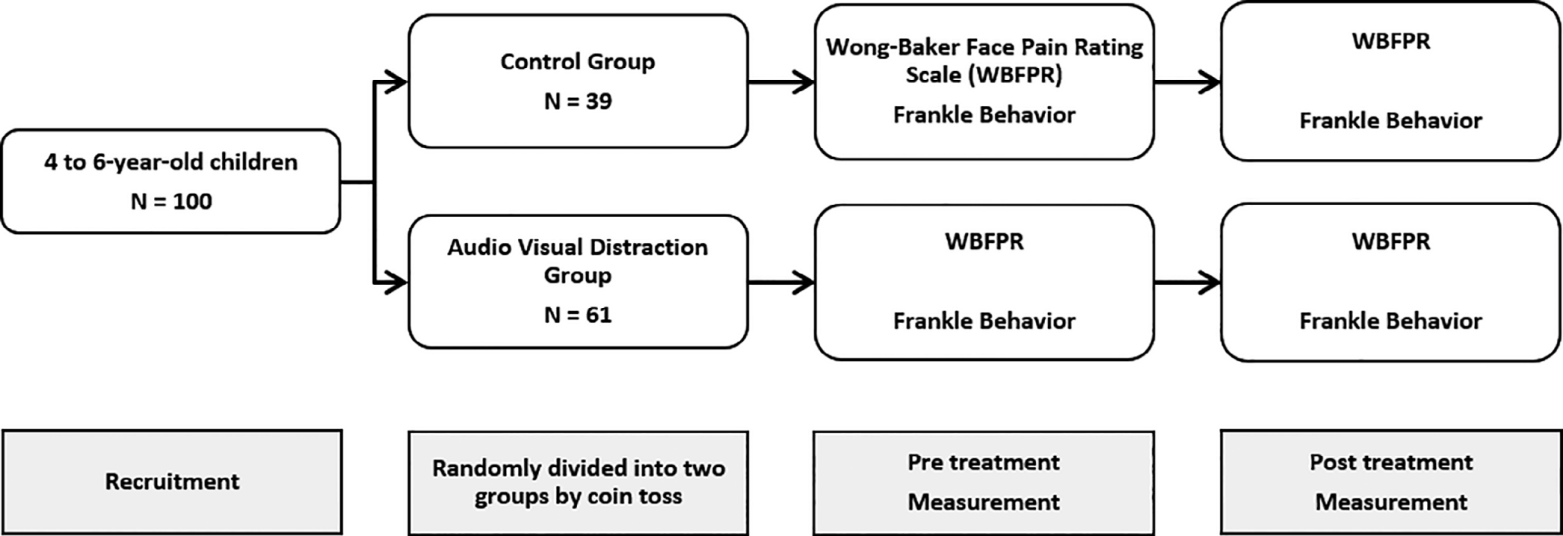
STROBE flow diagram: Participant recruitment for a single visit study

The University of Alabama at Birmingham (UAB) Institutional Review Board approved the proposed study (X161111003) and data was collected at the UAB Pediatric Dental Clinic from December 2016 to September 2017. Four to six‐year‐old children presenting with a parent to the pediatric dental clinic for dental check‐up with and without previous dental experience were recruited. Children were excluded from the study if they had significant cognitive or physical limitations or were accompanied by an adult other than their parents. If the child met the criteria, his/her parents were given the study information and invited to participate. The assent and consent for the study were attained following completion of child check‐in procedures and before dental treatment. All subjects were informed of the option to withdrawal from the study at any time without affecting their dental treatment. After the agreement of the participation, healthy 41 girls and 41 boys were enrolled and treated in the UAB Pediatric Dental Clinic (Figure 1). As standard dental procedure, medical and dental history were attained from each parent. During the history taking, child's experience of the previous dental visit was provided by their parents and noted as positive, neutral, and negative. Children were randomly divided into two groups by coin toss as a control (CTR, *N* = 39) group and audio visual distraction (AVD, *N* = 61) group.

### 2.2 Pain rating scales and behavioral rating scales

**FIGURE 2:**
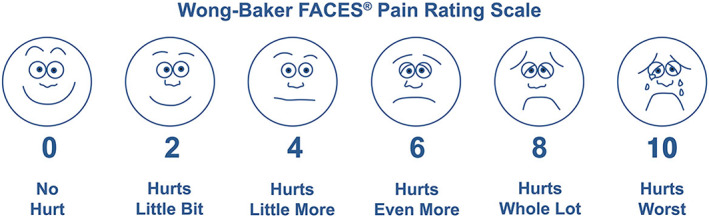
Wong‐Baker FACES® pain rating scale (Home ‐ Wong‐Baker FACES Foundation, n.d.). The Faces Pain Scale–Revised (FPS‐R) scored 0, 2, 4, 6, 8 and 10. It shows a close linear relationship with visual analogue pain scales across the age range of 4–6 years. It is easy to administer and requires no equipment except for the photocopied faces. Score the chosen face 0, 2, 4, 6, 8, or 10, counting left to right, so “0” = “no pain” and “10” = “very much pain.” Do not use words like “happy” and “sad.” This scale is intended to measure how children feel, not how their face looks. Wong‐Baker FACES Foundation (2018). Wong‐Baker FACES® Pain Rating Scale. Retrieved Oct 19, 2018 with permission from http://www.WongBakerFACES.org

One operating dentist was designated throughout the study to collect child's face pain perception scale and behavior scale. The descriptions are as follow: (1) The child's subjective pain expressions were collected by showing the Wong‐Baker Faces Pain Rating Scale (Home ‐ Wong‐Baker FACES Foundation, n.d.) panel to children (Figure 2), which was created for children to assist them communicate about their pain and used with children ages 3 and older (Wong & Baker, 1988). The Wong‐Baker Faces Pain Rating Scale is self‐assessment tool (0: No Hurt, 2; Hurts Little Bit, 4; Hurts Little More, 6; Hurts Even More, 8; Hurts Whole Lot, 10; Hurts Worst); therefore, the individual should be communicable to address own pain. (2) Dentist's perceptive determination of child's behavior was collected using the Frankl behavior rating scale following the American Academy of Pediatric Dentistry Behavior Guidance for the pediatric dental patient. (AAPD, 2020). The Frankl behavior rating scale is a frequently used behavior rating systems in both clinical dentistry and research. This scale indicates observed child's behaviors into four categories (1; Definitely negative, 2; Negative, 3; Positive, 4; Definitely positive).

### 2.3 Measurements

**FIGURE 3:**
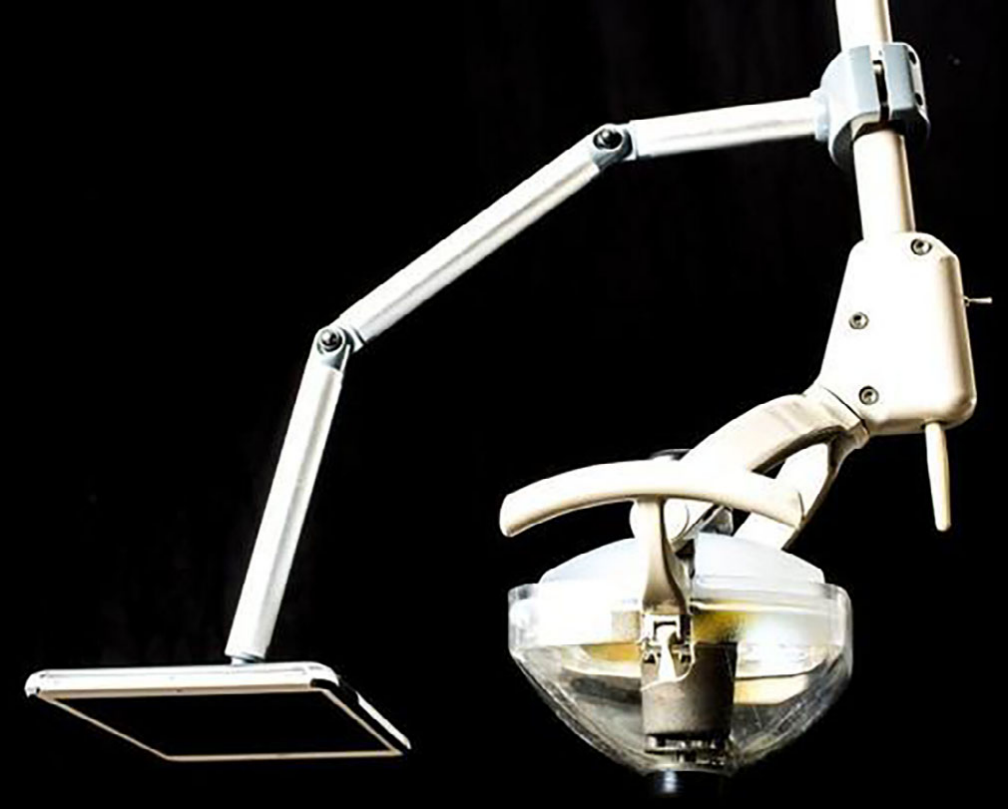
Molar media mount (Molar Media Mount, LLC, n.d.). The Molar Media Mount is made of lightweight, high‐strength materials, and is specifically constructed to work with device of 16 oz. or less in weight. The Molar Media Mount was used to mount the AVD and attached to the light compartment of dental chair unit with adjustable arm extension. The image used by courtesy from the Molar Media Mount

Prior to the dental procedure, each child from the both CTR and AVD groups was shown the Wong‐Baker Face Pain Rating Scale panel and pointed out their expression ranged as 0 (No Hurt) to 10 (Hurts Worst) as an initial evaluation. After the informed agreement of dental procedure, parents were asked to stay in the reception area during the dental procedure for both control and treatment groups. The CTR group received dental treatment under the basic behavioral guidance of TSD and Nitrous oxide (N_2_O) inhalation. The N_2_O inhalation procedure was provided as minimal sedation and followed by the guideline (Use of nitrous oxide for pediatric dental patients, 2017). The treatment group received dental treatment using the AVD. Children in both groups underwent an operative appointment either a placement of stainless‐ steel crown (SSC) restoration or an extraction under the administration of local anesthesia. The AVD was provided in the form of Disney copyrighted movies in English or Spanish version based on the children's preference. The AVD was attached to the dental light compartment using the extensive arm (Molar Media Mount LLC, Millcreek, Washington United States) as shown as Figure 3 (Molar Media Mount, LLC, n.d.). The mounted AVD is a 9.7‐in. wide screen and weighs 15.4 ounces. The device was able to be adjusted in vertical and horizontal direction to match each child's focus and inter‐pupillary distance. The Umbrella License was obtained from The Motion Picture Licensing Corporation to present the Disney movies to children in the pediatric clinic. The audio volume was adjusted to allow children to listen to dentist's instructions. Children of the AVD group selected and watched the movie during the entire dental operative procedure. Particularly, the movie was shown before the local anesthetic administration. The local anesthetics were provided as topical 20% Benzocaine followed by 2% Lidocaine infiltration (1:100,000 epinephrine) based on the child's weight for both AVD and CTR groups. The administered volume of local anesthetics was recorded in the child's electronic dental charts. Upon completion of the planned dental procedure, the pediatric dentist collected the children's face pain scale again using the Wong‐Baker Face Scale panel and assessed Frankl Scale during the procedure. In addition, the children were asked their satisfaction of the AVD while they received dental treatment and for the record.

### 2.4 Statistical analysis

The primary statistical analysis used Chi‐square or Fisher's exact tests to compare proportions of children with improved, unchanged or worsen in pain scores between the groups. The Mantel–Haenszel (MH) chi‐square statistic was used to compare ordinal post‐ treatment pain scores and Frankl scale scores between the groups. McNemar's test was used to compare pre‐treatment versus post‐treatment pain scores and pain score categories within each of the groups. Secondary analyses were conducted using multiple logistic regression to evaluate whether potential associations between child characteristics, previous dental experience and procedure type affected the comparisons of post‐treatment pain or Frankl scale scores between the study groups. For these analyses, pain scores were dichotomized into categories representing no pain versus any pain reported. Each model included main effect terms representing one child characteristic and study group, along with the two‐factor interaction term for characteristic by study group. The statistical test that is of primary interest in these analyses is the interaction term, as significance for this test would indicate that the association between the child characteristic and post‐treatment pain differs between the study groups, adjusting for presence of pre‐treatment pain.

## 3 RESULTS

Recruited child's demographic data stratified by CTR and AVD groups is shown in Table 1, including age, gender, race, previous dental experience, and dental procedure. Table 2 shows associations between previous dental experience and child characteristics. Notably, negative previous dental experience differs significantly associated between extraction versus SSC restoration procedures (*p* = 0.04). Other demographic data did not show significant associations with previous dental experience.

**TABLE 1:** Demographics and characteristics of participants by groups

	AVD^a^	CTR^b^	Total
Age	N (%)	N (%)	
4	18 (30)	14 (36)	32
5	23 (38)	13 (33)	36
6	20 (33)	12 (31)	32
Gender			
Female	31 (51)	20 (51)	51
Male	30 (49)	19 (49)	49
Race/Ethnicity			
African American	18 (30)	12 (31)	30
Hispanic	34 (56)	19 (49)	53
White	9 (15)	8 (21)	17
Previous dental experience			
Negative	39 (64)	18 (46)	57
Positive	10 (16)	12 (31)	22
Neutral	12 (20)	9 (23)	21
Dental procedure			
Extract	15 (25)	15 (38)	30
SSC^c^	46 (75)	24 (63)	70

*Note*: Percentages may not add up to 100 due to rounding the numbers.

^a^ AVD = Audiovisual Distraction Group.

^b^ CTR = Control Group.

^c^ SSC = stainless steel crown.

**TABLE 2:** Associations between patient characteristics and previous dental experience

Previous dental experience				
	Negative (*n* = 57)	Neutral (*n* = 21)	Positive (*n* = 22)	*p* value^a^
Age, mean ± SD	5 ± 0.8	4.9 ± 0.8	5.2 ± 0.8	0.29
Gender				0.07
Female	33 (67.4)	6 (12.2)	10 (20.4)	
Male	24 (47.1)	15 (29.4)	12 (23.5)	
Race				0.77
African American	19 (63.3)	6 (20)	5 (16.7)	
Hispanic	30 (56.6)	10 (18.9)	13 (13)	
White	8 (47.1)	5 (29.4)	4 (23.5)	
Dental procedure				0.04*
Extraction	20 (51.3)	13 (33.3)	6 (15.4)	
SSC^d^	37 (60.7)	8 (13.1)	16 (26.2)	
Treatment				0.16
AVD^b^	39 (63.9)	12 (19.7)	10 (16.4)	
CTR^c^	18 (46.2)	9 (23.1)	12 (30.8)	

^a^ Multiple logistic regression analysis, p‐value for interaction term; *p < 0.05 was considered statistically significant.

^b^ AVD = Audiovisual Distraction Group.

^c^ CTR = Control Group.

^d^ SSC = stainless steel crown.

The Wong‐Baker Face Pain Rating Scale of the pre‐ and post‐treatment was collected and recorded as 0, 2, 4 scales as shown in the Table 3. The participants from both groups did not indicate the scales of 6, 8, and 10. The distributions of pain scores were not significantly different between the AVD and CTR groups. Then, the scale changes from the pre to the post treatment were further classified as categories of improved, worsen, and unchanged scales (Table 4). Post‐treatment pain was commonly rated as 0 on the faces scale, with 68.5% (42/61) of AVD group and 76.9% (30/39) of CTR group reporting no pain. In the AVD group, 29.5% (18/61) of child rated post‐treatment pain as 4, while in the CTR group, 15.4% (6/39) rated their pain as 4. One (1.6%, 1/61) from the AVD group and three (7.7%, 3/29) from the CTR group reported pain scale as 2. Overall, in the AVD group, 32.8% (20/61) reported having any pain before treatment, and 31.2% (19/61) reported any pain following treatment with no significant difference between the pre‐ and post‐ treatments (*p* = 0.5637). In the CTR group, any pain was reported by 15.4% (6/39) and 23.1% (9/39) with no significant difference between the pre‐ and post‐ treatments (*p* = 0.0833). The distributions of pain scores were not significantly different between the AVD and CTR groups (*p* = 0.2073).

**TABLE 3:** Pre and post dental treatment Wong‐Baker faces pain rating scale

	AVD^a^		CTR^b^	
Pain scale	Pre	Post	Pre	Post
0	41 (67)	42 (69)	33 (85)	30 (77)
2	5 (8)	1 (2)	2 (5)	3 (8)
4	15 (25)	18 (30)	4 (10)	6 (15)
McNemar's test:		*p* = 0.5637		*p* = 0.0833

*Note*: MH chi‐square = 1.59, *df* = 1, *p* = 0.2073.

^a^ AVD = Audiovisual Distraction Group.

^b^ CTR = Control Group.

**TABLE 4:** Change in Wong‐Baker face pain rating scale

Characteristics	Improved	Worsen	Unchanged	*p* value
	*n* = 5(%)	*n* = 12(%)	*n* = 83(%)	
Age, mean ± SD	5 ± 1	4.8 ± 0.8	5 ± 0.8	0.52
Gender				1
Female	3 (5.9)	6 (11.8)	42 (82.4)	
Male	2 (4.1)	6 (12.2)	41 (83.7)	
Race				0.2
African American	1 (3.3)	7 (23.3)	22 (73.3)	
Hispanic	4 (7.6)	4 (7.6)	45 (84.9)	
White	0 (0)	1 (5.9)	16 (94.1)	
Dental procedure				0.0009*
Extraction	3 (7.7)	10 (26.6)	26 (66.7)	
SSC	2 (3.3)	2 (3.3)	57 (93.4)	
Treatment				0.1532
AVD	3 (4.9)	7 (11.5)	51 (83.6)	
Control	2 (5.1)	5 (12.8)	32 (82.1)	
Behavior in Frank scale				0.06
1	0 (0)	0 (0)	0 (0)	
2	1 (20)	2 (40)	2 (40)	
3	2 (40)	2 (6.5)	27 (87.1)	
4	2 (40)	8 (12.5)	54 (84.4)	
Previous dental experience				0.61
Negative	4 (7)	5 (8.8)	48 (84.2)	
Neutral	1 (4.6)	3 (13.6)	18 (81.8)	
Positive	0 (0)	4 (19.1)	17 (81)	

*Note*: Mantel–Haenszel (MH) chi‐square statistic was used to analyze post‐ treatment pain scores and Frankl scale scores between the groups.

^*^ Denotes statistical significance at *p* < 0.05.

Multiple logistic regression modeling indicated that none of the child characteristic variables were associated with post‐treatment pain from each study group. Overall, Table 4 demonstrated there was no significant association between the child's facial pain rating scale changes and any demographic characteristics. Notably, the type of dental procedure is significantly associated with the changes of child's pain rating scales. There was not a significant difference between the study groups in the percentages of children showing improved, worsen, or unchanged in pre‐ to post‐treatment pain scales.

Pre‐ and post‐treatment Frankl behavior scores were recorded by the dentist and evaluated the changes of child's behavior as improved, unchanged, and worsen are presented in Table 5. There was a significant difference between the AVD and CTR groups based on Frankl scale scores (*p* < 0.0001). In the AVD group, 91.8% of children (56/61) were classified as “Definitely positive” versus 35.9% (14/39) in the CTR group. No children in the AVD group were classified as negative versus 10.3% (4/39) in the CTR group. Twenty‐one children in the CTR group (53.9%) were classified as “Positive,” versus 8.2% (5/61) in the AVD group.

**TABLE 5:** Changes on Frankl behavior ratings by group

	AVD	CTR	*p*‐value^a^
Improved	56 (92)	14 (36)	<0.0001
Worsen	0 (0)	4 (10)	
Unchanged	5 (8)	21 (54)	

^a^ Mantel–Haenszel Chi‐Square = 33.9, *df* = 1.

## 4 DISCUSSION

Dental anxiety is a complex reaction to the unknown danger which reflects on individual's behavioral, cognitive and physiological components (Kida Minja & Kokulengya Kahabuka, 2019). It is a common human response and often modifies individuals' daily hygiene routine and prompts them to avoid dental care leading to a high risk of oral disease (Sohn & Ismail, 2005). The complexity of dental anxiety is initiated by a multifactorial origin including, child's past dental experience, pain, influence of family members, personality, and dental environmental aspect (sounds, unpleasant smell, local anesthetic injections) (Kida Minja & Kokulengya Kahabuka, 2019). With a strong association between anxiety and pain, such as needle insertion could amplify children's pain perception to dental treatment (Agarwal et al., 2017). In fact, dental anxiety may lead to delayed timely dental intervention and result in extensive dental treatments with high cost of full mouth rehabilitation. To manage children's dental anxiety, dentists communicate efficiently to provide the best options for the dental appointment (nitrous oxide inhalation, sedation, general anesthesia) and consider the overall aspect of behavioral guidance including, allocation of time and utilizing an effective distraction tool (Wells et al., 2018).

The study population was designated by age group of four to six‐year‐old children to minimize the communicational variable since under 4 years of pre‐school age group has less cognitive and communicational ability accompanied with uncooperative and disruptive behavior (Newton et al., 2012). Besides, the selected age group possesses interactive and responsive behavior upon the dentist's distraction technique application (Dahlquist et al., 2009). Parents could stay without active participation in the communication between child and dentist during the procedure. Parental presence may affect the results of children’ facial expression scale in psychological comfort; however, it does not affect the Frankl scale of the dentist. Despite controversial suggestions of parental presence/absence to manage children's behavior, the dentists' authority is the key behavioral guidance during the dental procedures (Cox et al., 2011). Therefore, specially trained dentists are essential to modify children's behavior to improve the relationship. In addition, it has been shown that children expressed stressful and uncooperative behavior in proportion to the duration of dental procedure (Jamali et al., 2018). The proposed dental treatments were either extraction or SSC restoration which were longer than 30 min and lesser than 60 min in both AVD and CTR groups. The operative dental appointments were arranged for both AVD and CTR groups in the afternoon to standardize the visit time for all children and to eliminate the chance of missing their school responsibilities. Some families expressed that school attendance is crucial, therefore appointment times were respectfully accommodated (Pinkham et al., 2005). Thereafter, unexpected confounding factors influencing the study outcomes were controlled well.

Similar to the AVD, a study suggested that the use of the AV eyeglass system was shown to be more efficient than a regular television screen and that it also could be used instead of nitrous oxide sedation (Ram et al., 2010). When compared to similar behavior guidance techniques, such as music relaxation, storytelling, listening to the audio by headphones, playing video games, and watching television, the AV eyeglass system has been shown to minimize the children's anxiety toward dental treatment. Furthermore, the AV eyeglass system has been noted to enhance the children's cooperative behavior (Hoge et al., 2012), which is consistent with the results of this study. However, the AV eyeglass system might limit the dentist's chair side activity. Whereas the AVD which mounted to the dental light compartment would provide dentist better accessibility. In addition, study reported that the AVD technique demonstrated a significantly effective mode of distraction to manage anxious children compared to audio distraction only (Kaur et al., 2015). In order to initiate positive behavior of child sitting in dental chair, the AVD could be introduced with TSD and the combined AVD‐TSD would be beneficial to reduce child's anxiety (Khandelwal et al., 2018).

This study selected two evaluation methods of observation, Wong‐Baker Pain Rating Scale and Frankl scale, to assess child feeling and dentist determination, respectively. The Wong‐Baker Pain Rating Scale was used as a self‐report measure that, appropriately used with children, provides an immediate state of emotional expression toward dental treatment. It has been reported as a valid indicator of a child's pain experience (Buchanan & Niven, 2002). However, children have limited cognitive/linguistic skills and reflect their feeling subjectively (Aartman et al., 1998). Hence, the Frankl Scale was used to evaluate children's behavior during the dental treatment procedure, which reflects on the dentist's perspective (Venham & Gaulin‐Kremer, 1979). The results suggested that the AVD was not associated with children's perception based on the pain scale but was significantly associated with dentist assessment indicated by the Frankl behavior scales. The results are supported by other studies using other AVD leading to less anxiety (Filcheck et al., 2005). Despite the no change in child's facial expression, the AVD is evaluated as an effective distractive behavioral guidance technique leading young children to less discomfort and encouraging cooperation during the dental procedure. Regardless of the results, the AVD increases the child's attention to the device and increases satisfaction for most children during dental treatment. In addition, most of the parents and dental staff were highly accepted and satisfied with using the AVD (Ram et al., 2010). While the AVD technique is not meant to replace the trust‐building communication that is inherent to good child‐clinician relationships or to replace the use of nitrous oxide, the study supports the use of AVD during dental procedures building upon the dentist‐child–parent trust to enhance the positive attitude toward the dental experience.

### 4.1 Limitations

Due to the one operating dentist in the study, the study could not be performed as a blind study, rather the assessment of the Frankl scale was consistent by one designated dentist. In actual clinic procedure, the sound of the AVD was not loud enough to mask other dental operatory sounds (e.g., suction and high‐speed handpiece noise) and it could disturb children. However, the entire study was performed in the similar noise environment. The AVD may interfere with interaction and communication between the child and dentist, which may hinder a proper connection between the child and dentist. The difference in sample sizes for the two groups could somewhat reduce the power of the study. However, the group sizes were the result of unrestricted randomization by coin toss, and the allocation was retained. The study was limited to 100 healthy children; however, it could be extended to increase child numbers and include those with physical and mental challenges in order to evaluate an effective behavioral guidance technique.

## 5 CONCLUSION

The AVD may not affect children's cognitive and emotional pain expression toward dental procedure, however it would be beneficial to divert the patient's attention from the un‐pleasant procedure. However, the AVD could be a useful adjunctive device to get children's attention toward dental procedure and could be utilized in certain populations to increase their behavioral cooperation. Therefore, the AVD should be used as an adjunctive device along with well‐established behavior management guidance.

## CONFLICT OF INTEREST

The authors declare no conflict of interest and no financial connection with the manufacturers of Molar Media Mount LLC.

## AUTHOR CONTRIBUTIONS

All authors have made substantial contributions to conception and design of the study. Alicia Delgado has been involved in conceived ideas, data collection, data analysis, and drafting writing. Soo‐Min Ok, Donald Ho, Tyler Lynd, and Kyounga Cheon revised manuscript. Kyounga Cheon supervised the project, revised the writing, and provided resources.

## Supporting information


**Appendix S1.** Supplementary information.

## Data Availability

The data that supports the findings of this study are available from the corresponding author upon reasonable request.
